# From the Cochrane Library: Interventions for Pityriasis Rosea

**DOI:** 10.2196/45388

**Published:** 2023-06-05

**Authors:** Alejandra Méndez, Carly Stevens, Andrea Murina

**Affiliations:** 1 Indiana University School of Medicine Indianapolis, IN United States; 2 Department of Dermatology Tulane University School of Medicine New Orleans, LA United States

**Keywords:** pityriasis rosea, acyclovir, antiviral, randomized controlled trial, Cochrane, evidence-based medicine, systematic review

Pityriasis rosea (PR) is a benign, self-limited skin disease characterized by the eruption of multiple erythematous plaques on the trunk and proximal extremities. The proposed etiology links the eruption to the reactivation of human herpesvirus (HHV) 6 and 7 in the skin [1]. HHV-6 viral reactivation during pregnancy has been associated with adverse birth outcomes in the first trimester of pregnancy [2]. PR may be asymptomatic or mildly pruritic and usually resolves without treatment within 12 weeks. PR and PR-like eruptions may also occur after vaccinations, other infections, and medications [3]. Patients with severe symptoms or extensive skin involvement may seek medical treatments.

A 2007 Cochrane review [4] and its updated 2019 version [5] investigated the efficacy of treatments for PR. We aim to provide a summary of the findings from the systematic review in this letter. A total of 14 randomized controlled trials (N=761) assessed two primary outcomes: good or excellent rash improvement within 2 weeks (participant-rated) and serious adverse events. Secondary outcomes included resolution of pruritus within 2 weeks (participant-rated), reduction in pruritus score within 2 weeks (participant-rated), good or excellent rash improvement within 2 weeks (investigator-rated), improvement in quality of life (participant-rated), and minor participant-reported adverse events. Interventions included macrolide antibiotics (erythromycin, azithromycin, clarithromycin), acyclovir, phototherapy, corticosteroids, antihistamines, and a traditional Chinese medicine called potenline. A meta-analysis was performed using a random-effects model for studies with similar interventions and controls. Most studies were conducted in Asia with participants ranging from the age of 2 to 60 years and the study duration lasting between 5-26 months. Three studies were funded by pharmaceutical manufacturers. There were no studies on pregnant women.

Macrolide antibiotics are not recommended for the treatment of PR. Erythromycin was shown in a single clinical trial to significantly reduce the pruritus score compared to a placebo (*P*<.001) [5]. However, there was no difference in investigator-rated rash improvement, and more adverse events were reported with erythromycin. Azithromycin showed no difference in investigator-rated rash improvement versus placebo.

Acyclovir monotherapy may be useful for the treatment of PR. Compared to a placebo, participants on acyclovir experienced greater resolution of pruritus (*P*=.04) [5]. Three trials showed a significant improvement in investigator-rated clearance with acyclovir versus a placebo (*P*=.004) [5]. Acyclovir and standard care (calamine lotion and cetirizine) reduced pruritus score (*P*<.001) and resolution of pruritus (*P*=.02) compared to standard care alone [5].

Using the Grading of Recommendations, Assessment, Development, and Evaluations (GRADE) framework, there is moderate quality evidence to support the use of acyclovir monotherapy for the rash and pruritus of PR and low to moderate quality evidence for erythromycin to treat pruritus in PR. A range of acyclovir dosages has been used in studies with unclear evidence based on different regimens (Table 1). Antivirals for severe or recalcitrant clinical presentations of PR may be used in patients who fail standard care.

In general, the self-limited nature and overall low incidence of PR pose challenges to study designs. Additional high-quality studies are needed to determine if acyclovir or other antivirals are superior to supportive care.

**Table 1:** Comparison of acyclovir dose regimens.

Study	Comparison	Dose regimen	Participants, n	Outcome	RR^a^ or MD^b^ (95% CI)
Ehsani et al [6], 2010	Acyclovir vs erythromycin	800 mg 5×/day for 10 days	14	Resolution of pruritus within 2 weeks (participant-rated)	13.22^a^ (0.91-192.02)
Rassai et al [7], 2011	Acyclovir vs no treatment	400 mg 5×/day for 1 week	54	Good or excellent rash improvement within 2 weeks (investigator-rated)	2.92^a^ (1.51-5.66) 1.52^a^ (1.14-2.01)
Ganguly [8], 2014	Acyclovir vs vitamin C	800 mg 5×/day for 1 week	60	Good or excellent rash improvement within 2 weeks (investigator-rated)	2.6^a^ (1.54-4.4)
Das et al [9], 2015	Acyclovir + calamine lotion + cetirizine vs calamine lotion + cetirizine	400 mg 3×/day for 1 week	24	Resolution of pruritus within 2 weeks (participant-rated) Reduction in pruritus score within 2 weeks (participant-rated) Minor participant-reported adverse events	4.50^a^ (1.22-16.62) 1.26^b^ (0.74-1.78) 7.00^a^ (0.40-122.44) 2.00^a^ (0.21-19.23) 5.00^a^ (0.27-94.34) 3.00^a^ (0.13-67.06)
Singh et al [10], 2016	Acyclovir vs placebo	800 mg 5×/day for 1 week	27	Resolution of pruritus within 2 weeks (participant-rated) Good or excellent rash improvement within 2 weeks (investigator-rated) Minor participant-reported adverse events	0.34^a^ (0.12-0.94) 0.19^a^ (0.01-3.56) 0.31^a^ (0.01-7.02)

^a^RR: risk ratio.

^b^MD: mean difference.

## Abbreviations

GRADE: Grading of Recommendations, Assessment, Development, and Evaluations
HHV: human herpesvirus
PR: pityriasis rosea
